# Paclitaxel Coated Balloon vs. Bare Metal Stent for Endovascular Treatment of Symptomatic Vertebral Artery Origin Stenosis Patients: Protocol for a Randomized Controlled Trial

**DOI:** 10.3389/fneur.2020.579238

**Published:** 2021-01-18

**Authors:** Yabing Wang, Yan Ma, Peng Gao, Yanfei Chen, Bin Yang, Yao Feng, Liqun Jiao

**Affiliations:** Department of Neurosurgery, Xuanwu Hospital, Capital Medical University, Beijing, China

**Keywords:** drug-coated balloon, bare mental stent, vertebral artery origin stenosis, endovascular treatment, posterior circulation ischemia

## Abstract

**Background:** Stenting treatment for refractory symptomatic patients with vertebral artery origin stenosis (VAOS) is safe; however, there is a high rate of in-stent restenosis. Although drug-eluting stents can reduce the incidence of restenosis to some extent, there is still a risk caused by stent fracture. Drug-coated balloon (DCB) has been proven to reduce the rate of restenosis in peripheral and coronary artery disease. DCB can prevent inflammation caused by extraneous material stimulation and allow the subsequent treatment that is characteristic of “leave nothing behind.” The purpose of this trial is to compare the efficacy and safety of DCB and bare metal stent (BMS) in the treatment of VAOS.

**Method/Design:** This trial is a 1:1 randomized, controlled, multicenter, non-inferiority trial that compares the DCB to BMS in terms of angiographically assessed target lesion binary restenosis (≥50%) at 12 months in endovascular treatment of symptomatic patients with VAOS.

**Discussion:** A total of 180 patients with symptomatic VAOS who match the trial eligibility criteria will be randomized 1:1 to treatment with DCB (*n* = 90) or BMS (*n* = 90). An angiographic core laboratory-adjudicated target lesion binary restenosis (≥50%) at 12 months of follow-up was selected as primary efficacy endpoint to assess the DCB treatment effect. A clinical events committee will assess the safety endpoints of all-cause death, target vessel related transient ischemic attack and ischemic or hemorrhagic stroke events. A data safety monitoring board will periodically review safety data for subject safety, the study conduct, and progress. In this trial, randomization is only allowed after successful pre-dilatation. We anticipate that this trial will provide rigorous data to clarify whether DCBs are beneficial in patients with symptomatic VAOS.

**Clinical Trial Registration:** www.ClinicalTrials.gov, identifier: NCT03910166.

## Introduction

Posterior circulation ischemic strokes account for 35–45% of all strokes. Seventy percent of posterior circulation ischemic strokes are caused by artery-to-artery embolism. Due to hemodynamic disorders and other reasons, atherosclerotic stenosis (which is also the main cause of posterior circulation ischemia) is most likely to form at the origin of the vertebral artery (1).

Studies have shown that 9–33% of patients with posterior circulation ischemia have vertebral artery origin stenosis (VAOS) or occlusion (2, 3). Moufarrij et al. found that patients with symptomatic VAOS had a stroke recurrence rate of 25–35% in the subsequent 5 years without treatment, and the 5-year survival rate was 60% (4).

The treatment of symptomatic atherosclerotic VAOS includes control of risk factors as well as drug, open surgical, and endovascular therapy. Drug therapy is the basic treatment modality for symptomatic atherosclerotic VAOS. For patients with refractory symptoms, surgical options such as endarterectomy and carotid artery transposition are considered. However, open surgical therapy requires considerable technical skill and Ausman et al. reported mortality and morbidity rates of up to 8.4 and 13.3%, respectively, for open surgical treatment (5). Endovascular treatment for VAOS is more feasible and safe than open surgical therapy as the rates of morbidity and mortality have been reported to be as low as 3.3 and 1.5%, respectively (6).

Bare metal stents (BMS) and drug-eluting stents (DES) are used in endovascular treatment of VAOS. However, extremely variable rates of significant in-stent restenosis (ISR, ≥50% diameter stenosis) after placement of either BMS or DES have been reported in the literature. In 2011, Stayman et al. conducted a systematic review of the endovascular treatment of VAOS. The results showed that the restenosis rate of DES was significantly lower than that of BMS (11–30%) (7). In 2019, Damian et al. reported that there was no significant difference in ISR rate between BMS and DES (22.8 vs. 19.4%, *p* = 0.635) after at least 6 months of follow-up (8). Most DES available have a limited diameter (up to 4 mm); for this reason, it is not possible to randomize patients to BMS and DES groups for direct head-to-head comparisons.

Werner et al. found that the two primary reasons for restenosis were stent fracture (with a rate of 32.1%) and intimal hyperplasia (which occurred at a rate of 20.7%) (9). Once restenosis caused by stent rupture occurs, there is a risk of serious ischemic events and there is a high failure rate regardless of whether the endovascular technique or surgical treatment is used (10, 11). Therefore, the prevention of adverse events (AEs) caused by stent fracture after endovascular treatment of VAOS remains an issue to be resolved.

In recent years, drug-coated balloon (DCB) has been proven to be safe and efficacious in the treatment of coronary artery disease and peripheral artery disease. In peripheral artery disease, DCB angioplasty is significantly more efficacious than percutaneous transluminal angioplasty (PTA) (12–14). In the treatment of coronary artery disease, DCB was originally used in the management of ISR and it has been proven to be safe and effective in the treatment of *de novo* coronary lesions. DCB cannot only reduce the restenosis rate caused by anti-proliferative drugs, but also involves no permanent deployment of extraneous material. DCB could avoid the inflammation caused by extraneous material stimulation and allow the subsequent treatment that has the characteristic of “leave nothing behind.” Endovascular treatment of VAOS with DCB has been proven to be feasible and safe (15, 16). An unpublished pilot study showed that a series of paclitaxel coated balloon, ORCHID, and DHALIA (Acotec scientific, Beijing, China) paclitaxel-coated peripheral balloon catheter were effective in the endovascular treatment of VAOS. ORCHID and DHALIA were approved for the endovascular treatment of peripheral artery disease by the National Medical Products Administration in 2016.

In a previous randomized controlled trial (RCT) of femoropopliteal arteries, DCB was shown to effectively reduce late lumen loss (0.05 ± 0.73 mm vs. 1.15 ± 0.89 mm, *p* < 0.001) and binary restenosis rates (22.5 vs. 70.8%, *p* < 0.001) compared to PTA (14). The series of DCB is an over the wire balloon catheter with a paclitaxel load of 3 μg/mm^2^. The product specification is suitable for the vertebral artery, with a diameter of 3.0–5.0 mm with 0.5 mm increments and a length of 20–40 mm. The main difference between them is that they match guidewires of different diameters; ORCHID and DHALIA match 0.035 and 0.018 inch guidewires, respectively. The only stent available and approved for endovascular treatment of extracranial cerebral vessels in China is APOLLO^TM^ (MicroPort Medical, Shanghai, China), a BMS made of stainless steel, with specifications of 3.0–5.0 mm in diameter and 8–23 mm in length. Therefore, this product had to be made the comparator in the RCT.

### Hypothesis

We hypothesized that DCB is non-inferior to BMS with regard to target lesion binary restenosis (≥50%) rate in the endovascular treatment of VAOS (≥70%) at 12 months.

## Methods

### Trial Design

This trial is a 1:1 randomized, controlled, multicenter, non-inferiority trial that compares DCB to BMS in terms of angiographically assessed target lesion binary restenosis (≥50%) at 12 months in the endovascular treatment of symptomatic patients with VAOS. All the patients screened should have refractory symptoms of posterior circulation ischemia after best medical treatment. Patients in the screening phase will consent to the trial before the invasive angiography procedure. The inclusion and exclusion criteria are listed in Table 1.

**Table 1:** Inclusion and exclusion criteria.

**Clinical inclusion criteria**
• Aged between 18 and 80 years old
• Symptomatic VAO stenosis refractory to best medical treatment
• Score on the modified Rankin scale ≤ 3
• NIHSS ≤ 6
• Patients have signed informed consent
**Clinical exclusion criteria**
• In-stent restenosis in vertebral artery
• Non-atherosclerotic arterial stenosis
• Non-vertebral artery stenosis caused TIA or minor stroke
• Intracranial stent implantation within 12 months
• Intracranial hemorrhage occurred within 3 months
• Obvious thrombosis in brain vessel, patients received lysis or thrombectomy 24 h before surgery
• Active bleeding or coagulation disorders
• Serious liver/kidney damage, not suitable for routine surgical treatment
• Patients with severe liver and kidney injury not suitable for routine surgical treatment
• Myocardial infarction or extensive cerebral infarction occurred within 2 weeks
• Uncontrolled high blood pressure
• Complicated intracranial tumor, cerebral arteriovenous malformation, or intracranial aneurysm
• Potential sources of cardiogenic thrombosis, such as mitral stenosis, atrial septal defect, aorta or mitral valve replacement, left atrial myxoma, etc.
• Life expectancy shorter than 1 years
• Patients whit cognitive impairment or mental disorders
• Known hypersensitivity to aspirin, heparin, clopidogrel, paclitaxel, contrast medium, etc.
• Pregnant and lactating women
• Patients who have participated in other clinical trials during the same period that lead to researchers who believe that patients may not be able to follow the trial program
**Angiographic inclusion criteria**
• The diameter of the normal segment of the artery beyond the stenosis between 3 and 5 mm, Target lesion has stenosis ≥70% evidenced by angiography
**Angiographic exclusion criteria**
• Severe calcified lesion or residual stenosis ≥30% after predilatation or flow-limiting dissection
• Tortuous or variable vessels
• Distal serial stenosis or distal vascular dysplasia of the stenosis segment

**Figure 1:**
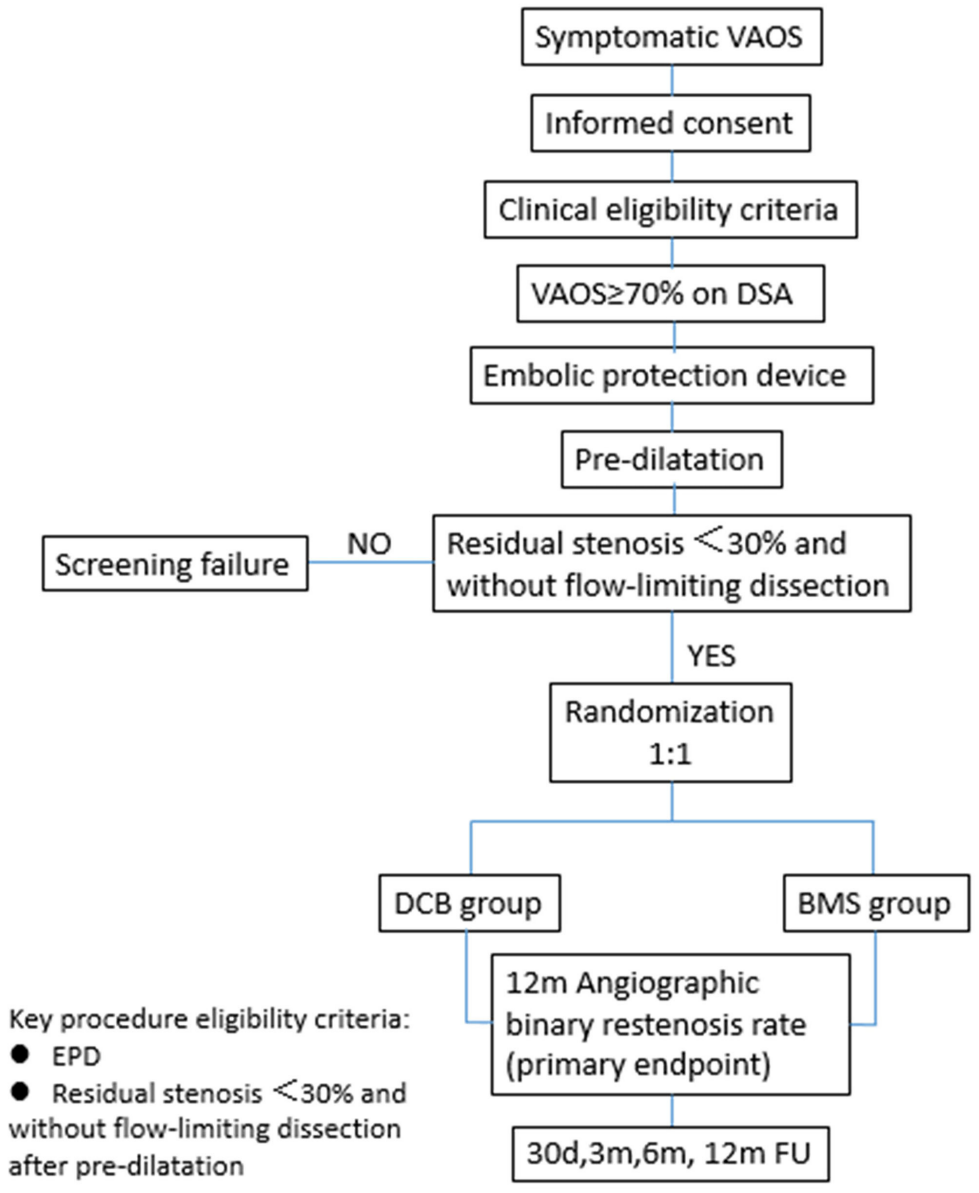
Randomization flowchart.

Patients who meet the angiographic criteria should undergo embolic protection device (EPD) placement into the distal segment of the target vertebral artery first, followed by pre-dilatation. The patients will be randomized to the DCB or BMS group after successful pre-dilatation, defined as residual stenosis <30% and without flow-limiting dissection. The flowchart of randomization is shown in Figure 1. The trial has been registered at clinicaltrials.gov (NCT03910166).

### Subjects

A total of 180 patients with symptomatic VAOS who match the trial eligibility criteria will be randomized 1:1 to treatment with DCB (*n* = 90) or BMS (*n* = 90). Target lesion angiographic binary restenosis will be assessed at the 12-month follow-up. All subjects will be evaluated for 1 year.

### Eligibility Criteria

Subjects will be required to meet all clinical and angiographic eligibility criteria (Table 1) to be considered for the trial. Subjects who meet any item of the exclusion criteria will be excluded.

### Endovascular Treatment Procedure

Antiplatelet therapy with 75 mg/day clopidogrel and 100 mg/day aspirin should be started 4 days before the procedure. In the procedure of angiography and endovascular treatment, the heparin dose during the intervention is 0.5–0.6 mg/kg body weight and, if required, an additional half dose after 1 h. The recommended size of artery sheath catheter is 8 F and the femoral artery approach is preferred. After cerebral artery angiography, a guiding catheter of 8 F will be advanced to the subclavian artery and a distal EPD will be placed into the V2 segment. All treatment devices will be manipulated over the EPD wire.

In this trial, endovascular therapy has been specified to start with balloon pre-dilatation. A plain uncoated balloon will be used to complete pre-dilatation and the recommended size is close to the target vessel diameter. The recommended ratio of balloon and target vessel diameter is 0.8–1.0 and inflation for 30 s at 10–14 bar is recommended. Additional inflation of pre-dilatation will be allowed to obtain enough luminal gain before the next treatment step. We will wait at least 5 min before angiography to evaluate elastic recoil. The patients with successful pre-dilatation will be randomized to the DCB or BMS group. For patients who fail pre-dilatation [with stenosis >30% or flow-limiting dissection (National Heart, Lung, and Blood Institute grade C and above)], the subsequent treatment will be determined by the operator.

For the DCB group, the DCB diameter should be close to the target vessel diameter to ensure sufficient paclitaxel delivery to the vessel wall, the recommended ratio of DCB to the target vessel diameter is 1:1, and the location of DCB angioplasty should cover the pre-dilatation site completely to prevent geographic miss. The DCB inflation time should not be <60 s, and the recommended inflation pressure is 8–10 bar. If a flow-limiting dissection occurs after DCB angioplasty, an APOLLO stent should be used to complete bailout stenting therapy. Patients randomized to the DCB group and who undergo bailout stenting will be tracked in the DCB group.

For the stenting group, the size of stent selected is the same as that of the DCB and the deployment site should also cover the pre-dilatation site. In addition, the proximal edge of the stent should be placed into the subclavian artery for 2–3 mm to ensure that the vertebral artery origin is completely covered. The recommended deployment inflation pressure is 8–10 bar for 30 s. All patients will undergo dual antiplatelet therapy for 3 months after the procedure, followed by long-term monotherapy with either clopidogrel or aspirin.

### Primary Efficacy Endpoint

An angiographic core laboratory-adjudicated target lesion binary restenosis (≥50%) at 12 months of follow-up was selected as the primary efficacy endpoint to assess the DCB treatment effect. If target lesion revascularization occurs within 12 months post-procedure, angiography before the revascularization should adjudicate whether it was a case of restenosis. The secondary endpoints include: (1) A device success rate assessed on a single device basis. For balloon catheter, it is defined as the successful reach of a target lesion, dilatation without rupture, and withdrawal. For stent, it is defined as residual stenosis <50%, without ischemic or hemorrhagic stroke events of the target vessel supplying the area after stenting. (2) Ischemic and hemorrhagic stroke events in the posterior circulation within 12 months of follow-up. (3) Transient ischemic attack in the posterior circulation during 12 months of follow-up.

### Safety Endpoint

The safety endpoint is defined as a target vessel related ischemic or hemorrhagic stroke event, transient ischemic attack event, or death at 30 days post-procedure.

### Follow-Up

Clinical follow-up will proceed as shown in Table 2. All patients will undergo follow-up for 12 months.

**Table 2:** Trial assessment requirements.

	**Screening (−14**~**0 d)**	**Procedure/baseline (0 d)**	**Discharge (1–7 d)**	**30 days (30 ± 7 d)**	**3 months (3 m ± 30 d)**	**6 months (6 m ± 30 d)**	**12 months (12 m ± 60 d)**
Demography	+						
Medical history	+						
ICF	+						
Eligibility criteria	+	+					
Physical examination	+						(+)
Antiplatelet/anticoagulant treatment	+	+	+	+	+	+	+
Routine lab testing	+		+				
neurological examination	+		+				
mRS			+	+	+	+	+
NIHSS	+		+	(+)	(+)	(+)	+
MRI(DWI)	+		+	(+)			
Duplex ultrasound	(+)		(+)	(+)	(+)	(+)	(+)
Cerebral artery angiography		+					+
Operation and device use record		+					
Hospital FU Visits				(+)	+	+	+
Telephone FU				+			
Adverse event assessment		+	+	+	+	+	+

*(+), Non-mandatory; +, mandatory*.

### Sample Size

The total sample size for the trial was calculated as 180 subjects, which fully powers the primary efficacy endpoints of target lesion binary restenosis rate (80%) based on initial estimates of the restenosis rate of each group, DCB and BMS, randomized at 1:1. The non-inferiority margin in the trial is considered to be 15%.

### Statistical Analysis Protocol

The relevant regulations for statistical analysis are in line with the International Council for Harmonization of Technical Requirements for Pharmaceuticals for Human Use E9 regulations and the relevant requirements in the Biostatistics guidelines for Clinical Trials issued by the National Medical Products Administration. All randomized patients will be analyzed by intention-to-treat analysis. An as-treated analysis will assess the effect of protocol violations. At the same time, all statistical analysis processes are carried out strictly in accordance with the standard operating procedure of the Statistics Center.

### Randomization

Randomization of subjects will proceed after successful pre-dilatation of target VAOS, which is defined as residual stenosis <30% and without flow-limiting dissection. Patients who do not meet the criterion of pre-dilatation will not be randomized. The randomization process will be performed by each investigator in the online electronic case report form (e-CRF) system on site. Multicenter competitive randomized enrollment will be performed. The number of subjects enrolled at each site should not exceed 50% of the total sample size. The number of investigational sites should not exceed ten in China.

### Ethical Considerations

The present trial is conducted in accordance with the principles of the Declaration of Helsinki, ISO 14155 and the national medical devices clinical practice regulations. The ethics committee of the principal investigational site has approved the trial protocol, and the trial protocol will be approved at all investigational sites. The principal investigation site is Xuanwu Hospital and the ethics committee is located at 45 Changchun Street, Xicheng District, Beijing. Written informed consent will be obtained from all subjects before enrollment. Subjects and their treating physicians will retain the right to withdraw from the trial and all follow-ups at any time without prejudice.

### Safety and Quality Control

#### Data Safety Monitoring Board

A clinical events committee will assess the safety endpoints of all-cause death, target vessel related transient ischemic attack, and ischemic or hemorrhagic stroke events. A data safety monitoring board will periodically review safety data for subject safety, the study conduct, and progress.

### Adverse and Serious Adverse Events

AEs are defined as any untoward medical occurrence in a subject whether or not considered related to the study device that is identified or worsens during the trial. Serious AEs refer to events during clinical trials, such as the requirement of hospitalization, prolonged hospitalization, disability, effects on one's ability to work, endangerment of life or death, congenital malformations, etc. All suspected AEs will be recorded on the AE Log in the e-CRF. AEs do not necessarily have a causal relationship with the test products and any medical device in the process of clinical application. There may be some unforeseeable defects due to the restrictions imposed by the level of science and technology at that time, the limitation of trial conditions, and other factors. Therefore, all AEs need to be recorded.

### Standards and Procedures for Trial Termination

When faced with any unexpected related serious AEs or any unexpected life-threatening situation in administering the test product, the test should be terminated immediately.

The trial can also be terminated in advance under the following circumstances:

- The sponsor proposed to terminate the trial
- The principal investigator, trial designer, or sponsor believes that the number of AEs makes it impossible to continue the trial
- If new data show that the emergence of research products raises safety concerns, continuing the trial may lead to unacceptable risks

Early termination of the trial must be subject to the written consent of the principal investigator and the sponsor.

### Data Collection

Data will be collected via the e-CRF during treatment at all investigational sites and will be completed prospectively during the hospital admission and follow-up.

## Discussion

We have presented the protocol of a multicenter RCT to assess whether DCB is non-inferior to BMS regarding target lesion binary restenosis (≥50%) rate in the endovascular treatment of VAOS (≥70%) at 12 months. In the present trial, randomization is only allowed after successful pre-dilatation. There are some considerations about the pre-dilatation requirement: (1) Obtaining a large lumen by pre-dilatation may reduce the binary stenosis rate (17, 18), (2) In addition, obtaining a large lumen can facilitate DCB to render a more uniform release of paclitaxel into the vessel wall (19, 20), which could impact on the incidence of restenosis, (3) The vessel contains smooth muscle and elastin, which leads to recoil, and lesions with high residual stenosis may not be applicable to DCB treatment. In conventional VAOS stenting operation, there is no mandatory pre-dilatation requirement. Therefore, we will clarify the requirement of pre-dilatation in the trial and in real-world practice, the key procedure step in the endovascular treatment of VAOS with DCB is pre-dilatation.

We anticipate that this trial will provide rigorous data that will clarify whether DCB are beneficial in patients with symptomatic VAOS.

## Ethics Statement

The studies involving human participants were reviewed and approved by the ethics committee of Xuanwu Hospital, the principal investigational site, and the trial protocol will be approved at all investigational sites. Written informed consent will be obtained from all subjects before enrollment.

## Author Contributions

YW: contributed to the original draft. LJ: conceived and designed the research. YC, BY, and PG were consulted about clinical issues. YM and YF were responsible for the revision of the draft. All authors contributed to the article and approved the submitted version.

## Conflict of Interest

The authors declare that the research was conducted in the absence of any commercial or financial relationships that could be construed as a potential conflict of interest.
